# Comparative Study of *Erythrina indica* Lam. (Febaceae) Leaves Extracts for Antioxidant Activity

**DOI:** 10.4103/0975-1483.62216

**Published:** 2010

**Authors:** SS Sakat, AR Juvekar

**Affiliations:** *Department of Pharmaceutical Sciences and Technology, Institute of Chemical Technology (ICT), Nathalal Parikh Marg, Matunga (E), Mumbai - 400 019, M.S, India*

**Keywords:** Antioxidant, *Erythrina indica*, gallic acid, polyphenols, radicals, rutin

## Abstract

The present study was designed to investigate the antioxidant activity of aqueous and methanol extracts of *Erythrina indica* Lam leaves by *in vitro* methods viz. 1, 1-Diphenyl-2-Picrylhydrazyl, nitric oxide radical scavenging activity, and inhibition of lipid peroxidation by thiobarbituric acid reactive substances (TBARS) method on isolated rat liver tissues. Quantitative analysis of antioxidative components like total amount of phenolics, flavonoids, and flavonols were estimated using the spectrophotometric method. Linear regression analysis was used to calculate the IC_50_ value. Results showed that the aqueous and methanol extracts exhibited significant DPPH radicals scavenging activity with an IC_50_ value 342.59 ± 19.59, 283.24 ± 12.28 µg/mL respectively. Nitric oxide radicals were significantly scavenged by the aqueous and methanol extracts (IC_50_ = 250.12 ± 10.66; 328.29 ± 3.74 µg/mL). Lipid peroxidation induced by the Fe^2+^ was inhibited by the aqueous extract with low IC_50_ value (97.29 ± 2.05 µg/mL) as compared to methanol extract (IC_50_ = 283.74 ± 5.70 µg/mL). Both the extracts were exhibited similar quantities of total phenolics. Total flavonoids were found to be in higher quantities than total flavonols in aqueous extract as compared to methanol extract. From the results, it is concluded that the aqueous and methanol extracts of *E. indica* leaves possesses significant antioxidant activity that may be due to the presence of flavonoids and related polyphenolic compounds.

## INTRODUCTION

*Erythrina indica* Lam (Febaceae) is a middle-sized quick growing tree found in Bengal and many parts of India especially in southern India. It is commonly known as ‘*Mandara*’ in Hindi and ‘*Indian coral tree*’ in English. It grows up to 18 m in height, the leaves are trifoliolate, β owers are borne in dense racemes, coral red and used traditionally for the treatment of liver trouble, joint pain, dysentery, convulsion, as a diuretic, laxative, and an anthelmintic.[1–3] Its powered bark is used in Indian folk medicine for rheumatism, itching, burning sensation, fever, asthma, and leprosy.[4] Aqueous extract of Sri Lankan *E. indica* leaves are reported to exhibit sedative but no analgesic activity.[5] It is also reported to exhibit anti-diuretic activity.[6]

Seeds of the *E. indica* showed the presence of flavone glycoside 5, 7, 4’-trihydroxy-3’-methoxy-8-C- prenyl flavone7-O-β-D-glucopyranosyl-(1→3)-α-L-arabino pyranoside.[7] Isoflavonoids such as 5, 4’-di-O-methylal pinumisoflavone, Cajanin, Indicanine B, and Indicanine C were isolated from the root bark of *E. indica*.[8]

Antioxidant activity has been proposed to play roles in various pharmacological activities such as anti-aging, anti-inflammatory, anti-atherosclerosis, and anti-cancer activities.[9,10] Inhibition of free radical induced damage by supplementation of antioxidants has become an attractive therapeutic strategy for reducing the risk of diseases.[11] Among the activities claimed to be present in most of the nutraceuticals and cosmeceuticals is the antioxidant activity. Several synthetic antioxidants are available, but are quite unsafe and their toxicity is of concern.[12] Natural products with antioxidant activity may be used for human consumption because of their safety. Hence, the present work was undertaken to investigate the antioxidant activity of aqueous and methanol extracts of *E. indica* Lam leaves using different *in-vitro* techniques.

## MATERIALS AND METHODS

### Plant material

The fresh leaves of *E. indica* Lam. were collected from the mature plant in and around the city of Mumbai, Maharashtra, India, during the month of August 2008 and dried under shade. The plant was authenticated by Dr. Ganesh Iyer, Botanist, Ramnarayan Ruia College, Matunga, Mumbai. A voucher specimen (2007/08/07) has been kept in our laboratory for future reference.

### Preparation of plant extracts

The dried powdered leaves of *E. indica* were defatted using petroleum ether (60-80°C) and successively extracted with methanol in the soxhlet extractor. Aqueous extract was prepared by the cold maceration method. Both the extracts were filtered through vacuum filter and the filtrate was concentrated in vacuum evaporator. Dried extracts were used for the further studies.

### Phytochemical evaluation

The aqueous and methanol extracts of *E. indica* leaves were studied for their phytoconstituents using different phytochemical tests.[13]

### Chemicals and reagents

1, 1-Diphenyl-2-Picrylhydrazyl (DPPH) was procured from Sigma Aldrich (St. Louis, MO, USA). Sodium nitroprusside (SNP) was purchased from Merck India Ltd., Mumbai. Orthophosphoric acid (H_3_PO_4_), perchloric acid, ferrous sulfate, sodium dodecyl sulfate (SDS), sodium nitrite (NaNO_2_), aluminium chloride (AlCl_3_), and Folin Ciocalteu’s reagent were purchased from S D fine Chem. Ltd, Mumbai. Thiobarbituric acid (TBA) was procured from Central Drug House Pvt. Ltd, New Delhi. N-1 Napthyletylenediamine dihydrochloride was obtained from LOBA CHEME Pvt. Ltd. Mumbai. All the other chemicals and reagents were of pure analytical grade and obtained from local supplier.

### Assessment of antioxidant activity

#### 1, 1-Diphenyl-2-picrylhydrazyl radical scavenging activity

The ability of the extracts to scavenge DPPH^.^ was determined by the method of *Gyamfi et al*.[14] with minor modifications. A 0.5 mL of aliquot of each extract at different concentrations (50-400 µg/mL) in methanol was mixed with 0.5 mL of 100 mM methanolic solution of DPPH. After 30 min incubation in darkness and at ambient temperature, the resultant absorbance was recorded at 517 nm. The percentage inhibition was calculated using the following formula.

$$\mathrm{Percentage}\;\mathrm{inhibition}\;=\;\left({\mathrm{Abs}}_{\mathrm{Control}}-{\mathrm{Abs}}_{\mathrm{Sample}}\right)\times 100/{\mathrm{Abs}}_{\mathrm{Control}}$$

IC_50_ values were calculated as the average of triplicate analyses

#### Determination of nitric oxide radical scavenging activity

The compound SNP is known to decompose in aqueous solution at physiological pH (7.2) producing nitric oxide radicals (NO ^.^). Under aerobic conditions, NO^.^ reacts with oxygen to produce stable products (nitrate and nitrite). The quantities of which can be determined using Griess reagent. The scavenging effect of the plant extracts on nitric oxide was measured according to the modified method of *Marcocci et al*.[15] 1 mL of extracts solution at different concentrations (25-400 µg/mL) were added in the test tubes to 1 mL of SNP solution (100 mM) and the tubes were incubated at 29°C for 2.5 h. An aliquot of 1 mL of the incubation solution was removed and diluted with 1 mL of Griess reagent (1% Sulfanilamide in 2% H_3_PO_4_ and 0.1% N-1-Naphthylethylenediamine dihydrochloride). The absorbance of the chromophore that formed during with Naphthylethylenediamine dihydrochloride was immediately read at 540 nm. The percentage inhibition was calculated using the formula mentioned above.

#### Inhibition of lipid peroxidation by thiobarbituric acid reactive substances method

Lipid peroxide formation was measured (lipid peroxidation assay) by the modified methods of *Ohkawa et al*.[16] and *Masao et al*.[17] Male Sprague-Dawley rat (weighing 200-250 g) was sacrificed by dislocation of the neck. The abdomen was opened; the liver was removed and homogenized in phosphate buffer saline (pH 7.0). Then, 1 mL of liver homogenate (10%, w/v) was added to the test extracts of different concentrations (25-400 µg/mL). The lipid peroxidation was initiated by adding 100 (L of 15 mM FeSO_4_ solution. After 30 min of incubation at room temperature, 0.1 mL of reaction mixture (liver homogenate + test drug) was taken in a tube containing 0.1 mL of SDS (8.1%w/v), 0.75 mL of 20% acetic acid, and 0.75 mL of 0.8% TBA solution. The volume in each tube was made to 2 ml with distilled water and then heated on water bath at 95°C for 60 min. After 60 min, the volume in each tube was made up to 2.5 mL and then 2.5 mL of N-butanol: Pyridine (5:1) was added in each tube. The reaction mixture was vortexed and centrifuged at 4000 rpm for 10 min. The organic layer was removed and absorbance was read at 532 nm in a UV spectrophotometer. The experiment was performed in triplicate. The percentage inhibition was calculated using the formula mentioned above.

### Quantitative analysis of antioxidative components

#### Determination of total phenolics, flavonoids, and flavonols

Total phenolics content were determined according to the method of Hammerschmidt *et al*.[18] Briefly, 0.2 mL of the test solution (10 mg/mL) was mixed with 1 mL of 10% Folin-Ciocalteu solution and 0.8 mL of 7.5% sodium carbonate solution. The mixture was incubated for 1 h at room temperature. The absorbance at 760 nm was measured and converted to phenolic contents according to the calibration curve of gallic acid.

Total flavonoids content was estimated colorimetrically based on the method modified by Zhishen *et al*.[19] To 0.1 mL of test extract (10 mg/mL) in a 10 mL volumetric flask, distilled water was added to make the volume to 5 mL and 0.3 mL 5% NaNO_2_ was added to this. 3 mL of 10% AlCl_3_ was added 5 min later. After 6 min, 2 mL of 1 M sodium hydroxide was added and the absorbance was measured at 510 nm. Rutin was used as a standard for constructing a calibration curve.

Total flavonols were estimated as rutin equivalents and expressed as mg of rutin per gram of dry extract by the method of Miliauskas *et al*.[20] The rutin calibration curve was prepared by mixing rutin solution with 2 mL (20 gm/L) AlCl_3_ and 6 mL (50 gm/L) sodium acetate. The absorption at 440 nm was read after 2.5 h at 20°C. The same procedure was carried out with 2 mL of plant extract (10 gm/L) instead of rutin solution. All determinations were carried out in triplicate and the mean values were used.

### Statistical analysis

The results are expressed as the mean ± SD for three replicates. Linear regression analysis was used to calculate the IC_50_ value.

## RESULTS AND DISCUSSION

### Phytochemical analysis

The phytochemical evaluation and extractive yield of the aqueous and methanol extracts of *E. indica* leaves are shown in Table 1. The aqueous extract revealed the presence of carbohydrates, proteins, glycosides, saponins, alkaloids, flavonoids, tannins, and phenolic compounds, while methanol extract showed the presence of carbohydrates, proteins, steroids, saponins, alkaloids, flavonoids, tannins, and phenolic compounds. Extractive yield of the aqueous and methanol extracts were found to be 14.26 and 7.89% w/w, respectively.

**Table 1:** Phytochemical evaluation of aqueous and methanol extracts of *E. indica* leaves

Phytoconstituents	Aqueous extract	Methanol extract
Carbohydrates	+	+
Proteins	+	+
Amino acids	−	−
Fats and oils	−	−
Steroids	−	+
Polysaccharides and glycosides	+	−
Saponins	+	+
Alkaloids	+	+
Flavonoids	+	+
Tannins and phenolic compounds	+	+
Extractive yield	14.26% (w/w)	7.89% (w/w)

+ = Present; − = Absent

### Assessment of antioxidant activity

#### 1, 1-Diphenyl-2-picrylhydrazyl radical scavenging activity

**Figure 1:**
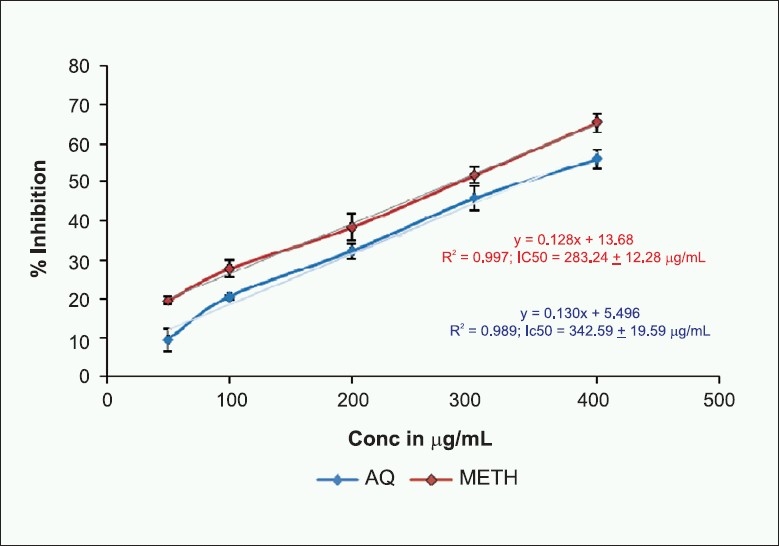
Effect of aqueous and methanol extracts of *E. indica* leaves on 1, 1-Diphenyl-2-picrylhydrazyl radical scavenging activity. Values represented in the figure are mean ± SD of three replicates; Linear regression analysis was used to calculate the IC_50_ value.

1, 1-Diphenyl-2-picrylhydrazyl radical scavenging assay is the most widely used method for screening antioxidant activity, since it can accommodate many samples in a short period and detect active ingredients at low concentration.[21,22] The decrease in absorbance of the DPPH caused by antioxidant was due to the scavenging of the radical by hydrogen donation. It is visually noticeable as a color change from purple to yellow. The aqueous and methanol extracts of *E. indica* showed DPPH radical scavenging activity in a concentration-dependent manner, with an IC_50_ value of 342.59 ± 19.59 and 283.24 ± 12.28 µg/mL, respectively [Figure 1].

#### Determination of nitric oxide radical scavenging activity

**Figure 2:**
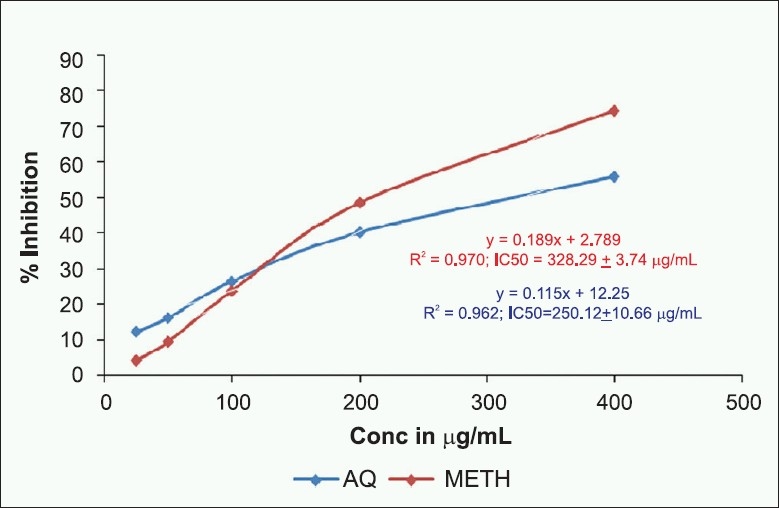
Effect of aqueous and methanol extracts of *E. indica* leaves on nitric oxide radical scavenging activity. Values represented in the figure are mean ± SD of three replicates; Linear regression analysis was used to calculate the IC_50_ value.

The effect of *E. indica* extracts on nitric oxide radical scavenging activity is shown in Figure 2. The compound SNP is known to decompose in aqueous solution at physiological pH (7.2) producing nitric oxide radicals (NO.). Under aerobic conditions, NO. reacts with oxygen to produce stable products (nitrate and nitrite). This leads to reduction of nitrite concentration in the assay media.[15] The aqueous extract of *E. indica* exhibited potent nitric oxide radical scavenging activity (IC_50_ = 250.12 ± 10.66 µg/mL) as compared with methanol extract (IC_50_ = 328.29 ± 3.74 µg/mL).

#### Inhibition of lipid peroxidation by thiobarbituric acid reactive substances method

**Figure 3:**
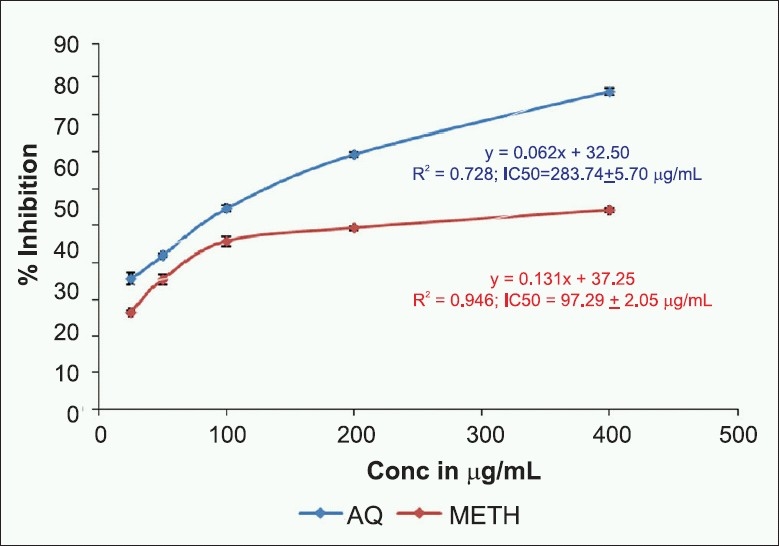
Effect of aqueous and methanol extracts of *E. indica* leaves on lipid peroxidation by TBARS method. Values represented in the figure are mean ± SD of three replicates; Linear regression analysis was used to calculate the IC_50_ value.

Lipid peroxidation is very important process in free radical pathology as it is damaging to cells. The liver of rat was used as a source of polyunsaturated fatty acids for determining the extent of lipid peroxidation. Malondialdehyde, a lipid peroxidation product, is an indicator of reactive oxygen species (ROS) generation in the tissue.[23] The inhibition of lipid peroxide formation by *E. indica* extracts is shown in Figure 3. The aqueous extract showed the maximum inhibition of peroxide formation with low IC _50_value of 97.29 ± 2.05 µg/ mL, whereas methanol extract showed IC_50_ value of 283.74 ± 5.70 µg/mL.

### Quantitative analysis of antioxidative components

#### Determination of total phenolics, flavonoids, and flavonols

The phenolic substances are known to possess the ability to reduce oxidative damage and act as antioxidants.[24] They can trap the free radicals directly or scavenge them through a series of coupled reactions with antioxidant enzymes.[25] In addition, it was reported that phenolic substances were associated with antioxidant activity and played important role in stabilizing lipid peroxidation.[26] This activity is believed to be mainly due to their redox properties, which play an important role in adsorbing and neutralizing free radicals, quenching singlet and triplet oxygen, or decomposing peroxides.[27] The aqueous and methanol extracts of *E. indica* showed the total phenol contents of 24.91 ± 0.00 and 25.62 ± 0.00 mg of gallic acid equivalents per gram of dry extract, respectively. Total flavonoids and total flavonols in aqueous extract were found to be 357.55 ± 33.38 and 265.14 ± 7.30 mg of rutin equivalents, respectively, whereas methanol extract showed total flavonoids 524.22 ± 16.17 and flavonols 167.21 ± 11.16 mg of rutin equivalents [Table 2].

**Table 2:** Quantitative analysis of antioxidative components of aqueous and methanol extracts of *E. indica* leaves

Antioxidative components	Aqueous extract	Methanol extract
Total phenolics	24.91 ± 0.00^a^	25.62 ± 0.00^a^
Total flavonoids	357.55 ± 33.38^b^	524.22 ± 16.17^b^
Total flavonols	265.14 ± 7.30^b^	167.21 ± 11.16^b^

a mg of gallic acid equivalent per gram of extract;

b bmg of rutin equivalent per gram of extract. Values represent in the results are mean ± SD of three replicates

## CONCLUSION

From the results of the study, it is concluded that the aqueous and methanol extracts of *E. indica* Lam. leaves possesses varying degree of antioxidant activity. The aqueous extract showed the potent antioxidant activity as compared with methanol extract. Further studies are required to identify the actual chemical constituents that are present in the crude extracts of this plant that are responsible for antioxidant activity.
